# A Self-management App for People Living With Mild Dementia (PRIDE): Protocol for a Pre-Post Feasibility Study

**DOI:** 10.2196/33881

**Published:** 2022-07-27

**Authors:** Abigail Rebecca Lee, Orii McDermott, Boliang Guo, James Roe, Martin Orrell

**Affiliations:** 1 Mental Health and Clinical Neurosciences Academic Unit School of Medicine, Institute of Mental Health University of Nottingham - Jubilee Campus Nottingham United Kingdom

**Keywords:** dementia, protocol, self-management, quality of life, web-based, psychology, social, intervention, app

## Abstract

**Background:**

With the rapid increase in the prevalence of dementia in the United Kingdom and beyond, the emotional, social, and economic burden on individuals, families, and health care services continues to rise. Currently, interventions that enable people living with dementia to better manage their condition and achieve a good quality of life are needed.

**Objective:**

This study aimed to explore how the Promoting Independence in Dementia (PRIDE) app can promote and support the self-management of people living with mild dementia.

**Methods:**

Feasibility of a pre-post study design incorporating the Reach, Effectiveness, Adoption, Implementation, and Maintenance framework will be studied. We will use up to 6 National Health Service Trusts as research sites and the Join Dementia Research website and accept self-referrals to recruit 60 to 90 people living with mild dementia. Participants will complete the PRIDE app intervention over 8 weeks with support from a dementia adviser facilitator. Measures exploring mood, physical well-being, and quality of life will be collected at baseline and at follow-ups at 3 and 6 months. Facilitators and National Health Service staff will be invited to complete interviews shortly after the intervention phase.

**Results:**

Data collection began in June 2021 and is predicted to cease by the end of August 2022. Analysis of the quantitative measures will explore the impact of the PRIDE app on participants’ independence, mood, and quality of life. Interview data will discuss participant experiences, how the use of the app affected them, and if it has the potential to be successfully implemented and maintained in dementia services.

**Conclusions:**

This study will show the potential reach, effectiveness, and adoption of the PRIDE app intervention in the lives of people with mild dementia. The findings from this study will inform future research on the PRIDE app and any further developments to improve its effectiveness.

**International Registered Report Identifier (IRRID):**

DERR1-10.2196/33881

## Introduction

### Background

Dementia currently affects an estimated 885,000 people in the United Kingdom [1]. Common symptoms include impaired ability and performance across multiple cognitive domains, such as memory, cognitive ability, and communication, which appear even in the early stages of the condition and can disrupt day-to-day activities [2-4].

Enabling people with dementia to achieve a high quality of life and live well independently has been highlighted as a priority in the United Kingdom [5]. With the increasing prevalence of dementia, providing people with the skills and understanding to manage their condition more effectively is now more important than ever. Given the progressive nature of dementia, self-management can support people with dementia and their families and optimize the level of autonomy and independence they are capable of and reduce excess disability [5]. Effective self-management of dementia requires individuals to understand their diagnosis and learn strategies to cope with the challenges that dementia brings [6]. This approach can help people retain independence and engage in daily activities and social relationships [6]. Self-management has the potential to benefit both the population with dementia and the health and social care sectors, as it supports individuals in maintaining their independence, increasing their overall well-being, and reducing the financial and social costs of paid and unpaid care [7]. Systematic reviews have suggested that psychosocial interventions have the potential to positively impact cognitive function, activities of daily living, and reduce disability [4].

Promoting Independence in Dementia (PRIDE), created by a team at University College London, is a psychosocial intervention for people living with mild dementia [8]. Delivered through a handbook and 3 facilitated sessions, PRIDE aims to improve the independence and quality of life of people with mild dementia and the friends and family that support them by enhancing decision-making, reducing stigma, and encouraging participation in mental, physical, and social activities. Elements of self-management were incorporated into PRIDE to encourage a person with dementia to take an active role in managing their condition [9]. For PRIDE, the selective optimization and compensation model was identified as a suitable approach, as it encourages giving the individual as much control as possible [9]. The selective optimization and compensation model proposes that people can manage their lives independently and successfully through these 3 regulations [10]. Selection focuses on identifying strengths and goals, optimization makes the best use of resources to maintain a person’s independence, and compensation finds alternative ways or external aids to adapt and promote engagement [9,10]. As part of the PRIDE feasibility study, the research team transformed the information from the paper handbook into a web-based platform, the PRIDE app [8]. During this phase, patient and public consultations were conducted on the initial prototype app to support the adaptation from paper to a web-based format. For this study, researchers at the University of Nottingham (UoN) have worked closely with the app development company to further enhance this PRIDE app prototype, which is now ready to be piloted by participants. Feedback from this study will contribute to future developments in the app to increase its usability. In this context, the term *app* refers to the intervention being web-based, rather than a downloadable app, and accessible on computers, tablets, and mobile phones

This study will incorporate the Reach, Effectiveness, Adoption, Implementation, and Maintenance (RE-AIM) framework [11] to explore the effectiveness and impact of the PRIDE app on people living with mild dementia. The RE-AIM framework was designed to assess and evaluate health behavior interventions, better understand their impact, and improve the translation of research into broader health services [11]. It has been successfully incorporated into the design, reporting, and reviewing of other self-management–focused studies, trialing web- or app-based interventions [12,13]. One example was demonstrated by Yoshida et al [13] who incorporated RE-AIM to review app- and text messaging–based self-management interventions in diabetes. The reporting of factors varied between the dimensions within the 20 included studies. Factors of reach (inclusion and exclusion criteria, sample size, and participation rate), effectiveness (results of follow-ups), adoption (description of intervention location), and implementation (intervention duration and frequency) were reported in the included papers [13]. However, there was a lack of reporting on some factors, including representativeness (reach), attrition rates (effectiveness), description of staff who delivered interventions and the method used to identify and recruit them (adoption), cost of implementation measures (implementation), and cost of maintenance measures (maintenance). Overall, many gaps were identified in the reporting of RE-AIM criteria in mobile-based intervention studies, which need to be resolved through further research to improve the quality of reporting [13]. The RE-AIM framework is constructed using five dimensions:

1. Reach—whether an intervention found the target population
2. Effectiveness (or Efficacy)—the short- and long-term impacts of an intervention
3. Adoption—whether the target staff, settings, and individuals use the intervention
4. Implementation—whether the intervention has been delivered and implemented as intended
5. Maintenance—the degree to which an intervention is sustained over time and in the most cost-effective manner

### Objectives

This protocol is written in accordance with the SPIRIT (Standard Protocol Items: Recommendations for Interventional Trials) checklist for reporting protocols [14]. The overall aim of the study is to explore how the PRIDE app can support the self-management of people living with mild dementia, using the RE-AIM framework: (1) the extent to which the PRIDE app has the capacity to reach people with mild dementia, (2) the effectiveness of the intervention, and (3) the adoptability of the intervention. The findings will contribute to future developments of the PRIDE app and inform a larger trial of its effectiveness.

## Methods

### Ethics Approval

This study has been reviewed and approved by the Oxford Research Ethics Committee (21/SC/0066). All minor and substantial amendments will be reviewed by the UoN and Oxford Research Ethics Committee. All participants, supporters, and interviewees will provide written informed consent.

### Study Design

We plan to conduct a pre-post feasibility study of the PRIDE app in people living with mild dementia. The RE-AIM framework [11] will enable us to identify key components for effective adoption, successful implementation, and sustained use of the PRIDE app, and identify potential barriers to the wider use of web-based psychosocial interventions for dementia.

The expected data collection period will be up to 12 months from the enrollment of the first participant. Participant recruitment will be carried out for up to 6 months, and follow-up will continue for a maximum of 6 months following the end of recruitment. All 5 RE-AIM framework dimensions will be explored in this study. However, as the intervention is not being implemented in normal routine care, the implementation and maintenance dimensions will not be assessed in depth and will instead be explored as secondary objectives.

### Study Setting

Research activities, including participant recruitment and intervention delivery, will be carried out within secondary care National Health Service (NHS) Trusts. The study will start as a single NHS Trust site, using relevant services within their region, and then proceed to recruit up to 5 additional research sites through the National Institute for Health and Care Research’s Clinical Research Network portfolio. To give sites more flexibility, the services they use are within their discretion. Any service with the capacity and where service users meet the inclusion criteria is eligible, and sites can use as many services as they have the capacity to. All intervention delivery and data collection activities will be conducted remotely, either on the web or via telephone or video calls.

### Recruitment

There will be 3 possible pathways through which potential participants will be identified for recruitment in the study.

#### NHS Pathway

Participants will be recruited from NHS Services for people with dementia within participatory care trusts by their research and delivery team. The initial approach will be from a member of the patient’s usual care team, who will obtain patients’ consent to pass their details onto the research and delivery teams, who will then complete a prescreening telephone interview and the case report forms.

Recruitment from this pathway will be divided into group targets, such as age and ethnicity, to increase the diversity and representativeness of the end participant sample. For example, recruiting participants will be divided into the following age groups: >65 years, 65 to 74 years, 75 to 84 years, and >85 years. The initial target will be to recruit 15 participants from each age group. Similarly, with ethnicity, the initial target will be to recruit a minimum of 1 Black, Asian, or Minority Ethnic individual for every 3 White participants. It is hoped that by using group targets, the recruited participants will represent the full spectrum of people living with mild dementia in England. If the ethnicity of participants is not as diverse as possible, then sites will be asked to oversample BAME to maximize their representativeness in the final participant group.

#### Join Dementia Research

Join Dementia is a web-based self-registration service that enables volunteers with memory problems or dementia, carers of those with memory problems or dementia, and healthy volunteers to register their interest in participating in research. We will register the study at the site and set inclusion and exclusion criteria. Volunteers who register their interest in the study will be contacted by the UoN team, who will then conduct the prescreening telephone interview and complete the case report forms.

#### Self-referral

Participants will also be able to self-refer directly to the UoN team. Potential participants may become aware of the study through relevant local and national charities, patient organizations, and through the general promotion of the study through relevant organizations’ newsletters, social media, mailing lists, and websites.

### Participants

The minimum recruitment aim for the entire study is 60 participants living with dementia and a maximum of 90 participants. The recruitment target for individual NHS Trusts will be 10 to 15 people living with dementia. Each participant will have the option to participate with a supporter (a relative or close friend); however, this is not a criterion for inclusion. All participants will be assigned to the PRIDE app, and they will continue to receive their usual care outside of the study. The ability to provide informed consent is vital. As we are unable to collect this in person owing to the impact of the COVID-19 restrictions, informed consent forms and information sheets were provided to interested participants. Members of the research or NHS site teams will go through the documents over telephone or video calls with everyone to ensure that they understand these documents before signing up for the study.

### Inclusion and Exclusion Criteria

The inclusion and exclusion criteria are presented in Textbox 1.

**Inclusion and exclusion criteria.**

**Inclusion criteria**

- Aged ≥18 years
- Self-report a medically confirmed diagnosis of mild dementia
- Able to provide informed consent and engage with the intervention
- Have access to Wi-Fi, a computer or tablet computer, telephone number, and email address.

**Exclusion criteria**

- Living in a care home or other institutionalized setting

### Intervention

#### Overview

The PRIDE app is a web-based handbook that provides information, case stories, and support for self-management across a range of topics often affected by a dementia diagnosis. The topics covered within the app are Keeping Mentally Active, Keeping Physically Active, Keeping Socially Active, Making Decisions, Getting Your Message Across, Receiving a Diagnosis, and Keeping Healthy.

This study will be delivered by facilitators called dementia advisers and PhD students managing the study. The advisers will usually be NHS workers, ideally with some prior experience in dementia services, who volunteer to complete 2 mandatory training sessions and can commit to delivering the intervention to at least one participant. Training sessions, delivered by the PhD student, will last 20 to 45 minutes and introduce facilitators to the PRIDE program and the key sections of the PRIDE app. Following training, dementia advisers will be paired with the participants and will begin the PRIDE app intervention. There will be 3 one-to-one sessions, delivered remotely via video or telephone calls, which will last between 30 and 90 minutes each and will be spaced 2 to 4 weeks apart.

#### Session 1: Introduction

Lasting approximately 60 to 90 minutes, this session will provide participants with a brief overview of the aims of PRIDE, complete the core introductory session pages, encourage them to reflect on their daily activities, and introduce the PRIDE app.

The general content of the Introduction session is presented in Textbox 2.

Advisers will encourage participants to identify important aspects of their daily lives, discuss how to maintain or enhance the activities or routines they value, and identify new activities they might benefit from. Participants will choose 3 topics and plan at least one activity they want to work on, which will be reviewed in later sessions.

**General content of the introduction session.**

1. Aim of Promoting Independence in Dementia (PRIDE)
2. Complete PRIDE profile
3. Core topics
  Finding a balance
  People and connections
  Keep going
4. Personalize topics—participants will choose 3 main topics to focus on
5. Familiarization with the PRIDE app
  Log-in process
  Adding social contacts
  Activity plans

#### Session 2: Review

The PRIDE app has a built-in review page for participants to complete alongside their advisers, and all key discussion points and progress will be recorded. Advisers will encourage participants to reflect on their progress and create or amend specific plans for activities or actions that will promote their independence. Choices and activities may be refined according to the participants’ and supporters’ experience of implementation and any needs that may have arisen since the first session. Barriers that may have prevented progress will be discussed, and the solutions will be explored. New activity options may also be set within the lifestyle domain topics. Emphasis will be placed on encouraging participants to continue implementing their plans between their sessions.

Session discussions will include the following:

1. Progress since the last session and providing positive feedback
2. What worked or helped them achieve goals and what hindered
3. Overcoming barriers
4. Satisfaction with current plans and if any changes are wanted

#### Session 3: Final

In the final session, participant progress will be reviewed again, and a maintenance plan exploring how PRIDE could continue to support them after the study will be developed to encourage long-term change.

Session discussion will include the following:

1. Progress since the last session
2. How PRIDE could continue to help them in the future—PRIDE’s “Plan, do, review” steps are a practical approach to help them continue their everyday activities
3. Encouragement to maintain a normal routine and social contact and use the steps when planning new activities

#### Plan, Do, Review

A principal technique of the PRIDE program is plan, do, review, and advisers will incorporate the technique to support participants in creating specific plans for activities or actions that will promote their independence. The participant and supporter will put their plans into practice between sessions and record their progress on the PRIDE app. To encourage participants, advisers will do the following:

1. Help them think about the action they would like to take or the activity they would like to do that would promote their independence
2. Support them in planning activities they would like to work on based on their topic choices, such as where their activity will take place, when they can begin their action plan or start making changes, and how they can do things in different ways
3. Explain how to record activities between sessions

### Evaluation Outcomes

This study will record quantitative and qualitative data to collect all aspects of the RE-AIM framework that we will explore. Table 1 outlines how each RE-AIM concept will be explored through analyses of quantitative and qualitative data.

**Table 1:** How Reach, Effectiveness, Adoption, Implementation, and Maintenance (RE-AIM) dimensions will be addressed in the study.

RE-AIM dimension	Definition	How addressed in the study
Reach	The absolute number, proportion, and representativeness of individuals contacted and those who are willing to participate in the intervention and reasons given as to why or why not choose to participate in the study.	Recruitment and characteristic figures (identification): eligibility rate, characteristics of eligible people approached (age, gender, and ethnicity), participation rate, and representativeness of participants; app use data, participant characteristics, and interviews (engagement): Did participants engage regularly with the PRIDE^a^ app? What were the characteristics of those who used the app and why? The baseline to 6-month participation figures.
Effectiveness	Does the PRIDE app positively impact important individual outcomes, such as mood and quality of life and whether there are any potential negative effects?	Change of pre- and postintervention scores: CASP-19^b^, IADL^c^, EQ-5D-5L, GDS^d^, EID-Q^e^, and global change measure.
Adoption	The absolute number, proportion, and representativeness of settings and the target patient group and intervention facilitators who are willing to initiate a program and why.	Postintervention qualitative interviews with participants: How did participants feel they benefited from using the app and why or why not? How did the app affect their lives; for example, impact on daily activities and independence? Did they need additional help to use it? app use: How much did participants use the app and for how long? Which elements were most useful? participant retention rate: How many participants continued the study after baseline? How many completed the 3 intervention sessions? interviews with facilitators and clinical staff: How would the app fit into the existing services? How well was it delivered? Who is best to deliver it? How will the app be paid for?
Implementation	The extent to which an intervention may be delivered as intended and whether individuals would use the intervention.	Postintervention qualitative interviews with participants, facilitators, and clinical staff (information on delivery, barriers for delivery, and implementation): the ease of using the app, whether workarounds were needed, and if so, why? How would the app fit into the existing services? Who is best to deliver it? How will the app be paid for?
Maintenance	The long-term effects of a program on outcomes (usually 6 or more months) and the extent a program becomes part of routine practice.	Postintervention qualitative interviews with participants, facilitators, and clinical staff: How would the app fit into the existing services? Who is best to deliver it? How could the app be integrated into the existing care system?

^a^PRIDE: Promoting Independence in Dementia.

^b^CASP-19: Control, Autonomy, Self-realization, and Pleasure Scale-19.

^c^IADL: Lawton Instrumental Activities of Daily Living Scale.

^d^GDS: Geriatric Depression Scale.

^e^EID-Q: Engagement and Independence in Dementia Questionnaire.

### Sample Size

For a pre-post comparison, 62 participants will be needed to detect a moderate effect size (Cohen *d*=0.4 and correlation=0.4) using 80% power at a 2-tailed .05 significance level. We will approach up to 200 people with mild dementia and aim to recruit a minimum of 60 and a maximum of 90 participants for the study, depending on the resources available, each with an optional supporter. These figures represent the total number of participants with dementia across all recruitment sites.

### Quantitative Outcomes

#### Overview

Quantitative measures will be collected at baseline, 3 months, and 6 months from participants and supporters. For participants, the outcomes collected will help to evaluate the effectiveness of the PRIDE app and its impact on their quality of life. Measures completed by supporters will explore the impact of the PRIDE app on their mood, quality of life, and perceived change in their relatives or friends with dementia. Measures will be completed either on the web or on paper, with the final decision left to the participant or the supporter. All participants and supporters will have the option to complete their questionnaires with the help of a researcher, who will be either a PhD student or a member of their local research team, and this will be done remotely over telephone or video calls. As measures will be completed remotely, the researchers will be reliant on the participants or supporters to communicate any difficulties encountered when completing them.

#### People Living With Dementia

##### Control, Autonomy, Self-realization, and Pleasure Scale-19: Baseline and 3 Months and 6 Months After the Intervention

The Control, Autonomy, Self-realization, and Pleasure Scale [15] has 19 items, each measured on a 4-point Likert scale (0=never, 1=not often, 2=sometimes, and 3=often). Items will include “I feel left out of things” and “I enjoy the things that I do.” Scores range from 0 to 57, with higher scores indicating higher levels of well-being [16]. The total and individual item scores will be recorded and used for the analysis.

##### EuroQoL Quality of Life Questionnaire-5 Domains, 5 Levels: Baseline and 3 Months and 6 Months After the Intervention

The EuroQoL Quality of Life Questionnaire-5 Domains, 5 Levels [17] measures 5 domains of quality of life: mobility, self-care, usual activities, pain or discomfort, and anxiety or depression. Each domain has 5 levels: no problems, slight problems, moderate problems, severe problems, and extreme problems. The levels are scored from 1 to 5 to indicate increasing severity. The participant indicates which level is most appropriate for their situation and provides a self-rated health score on the vertical visual scale, which ranges from 0 to 100 (where 100 is the best health). Individual item and health scores will be recorded and used in the analysis.

##### Lawton Instrumental Activities of Daily Living Scale: Baseline and 3 Months and 6 Months After the Intervention

The Lawton Instrumental Activities of Daily Living Scale [18] contains 8 domains that assess an individual’s ability to complete tasks necessary for independent living, such as preparing meals and maintaining a clean house. Each domain will be scored either 0 or 1, and a summary score of 0 (low functioning) to 8 (high functioning) will be used in the analysis. The measure is particularly good at identifying how a person is functioning at present and for identifying improvement or deterioration over time.

##### Geriatric Depression Scale: Baseline and 3 Months and 6 Months After the Intervention

The Geriatric Depression Scale (short form) is a 15-item measure that can be self-reported or read out to the participants if required. Each item has a “yes” or “no” answer, and the response indicating depression is scored as a point. A score of 0 to 5 is normal, a score >5 suggests depression, and a score of ≥10 indicates depression [19]. The total score will be used in the analysis.

##### Engagement and Independence in Dementia Questionnaire: Baseline and 3 Months and 6 Months After the Intervention

The Engagement and Independence in Dementia Questionnaire has 26 items that assess the degree to which a person with dementia feels independent and engages socially with those around them. It reflects the multifaceted nature of independence in dementia and includes items related to remaining active, decision-making, reciprocity, and connectedness to others. Each item is measured on a 5-point Likert scale (0=not true at all, 4=true nearly all the time) and was developed for a sample of older adults with dementia [20]. The total and individual item scores will be recorded and used for the analysis.

##### Global Change (Self-rated): 3 Months and 6 Months After the Intervention

The global change measure will ask participants about any change in their well-being and sense of independence since the baseline. The questions will be “Compared with 3/6 months ago when you started in the PRIDE study, how would you rate your general well-being now?” and “Compared with 3/6 months ago when you started in the PRIDE study, how independent do you feel now?” A 5-point ordinal scale (1=much better, 3=no change, and 5=much worse) and (1=much more independent, 3=no change, and 5=a bit less independent) will be used to measure change.

#### Supporters (Informal Carers)

##### General Health Questionnaire: Baseline and 3 Months and 6 Months After the Intervention

The General Health Questionnaire [21] has 12 items that assess an individual’s current state and asks whether it differs from their usual state. Each item is rated on a 4-point scale (less than usual, no more than usual, rather more than usual, or much more than usual). Two popular scoring methods are used: General Health Questionnaire (0-0-1-1) and Likert (0-1-2-3), each providing a total score for analysis. The total and individual item scores will be recorded for the analysis. For both methods, the wording of the items means that reverse scoring is not required. The severity level is indicated by how high the score is.

##### EuroQoL Quality of Life Questionnaire-5 Domains, 5 Levels: Baseline and 3 Months and 6 Months After the Intervention

As with the participants, individual item and health scores will be used in the analysis.

##### Global Change (Proxy-Rated): 3 Months and 6 Months After the Intervention

The global change measure asks supporters about their perceived change in participants’ well-being and sense of independence since baseline. The questions will be “Compared with 3/6 months ago when you started in the PRIDE study, how would you rate the general well-being of your relative/friend now?” and “Compared with 3/6 months ago when you started in the PRIDE study, how independent do you feel your relative/friend is now?” A 5-point ordinal scale (1=much better, 3=no change, and 5=much worse) and (1=much more independent, 3=no change, and 5=a bit less independent) will be used to measure change.

### Qualitative Outcomes

The experiences and perspectives of the study participants and facilitators will be explored through postintervention interviews. Up to 20 participants and dyads will be invited to discuss and reflect on their experience of using the PRIDE app. Additional interviews will be conducted with up to 10 facilitators and 5 senior NHS service staff to explore their experiences of delivering the intervention and how the PRIDE app could be implemented into existing services. Interviews and analysis will be conducted by a PhD student managing this study.

Participants and their supporters, if taking part, will be invited to attend an interview at 8 to 10 weeks, shortly after the completion of the intervention. The final number of interviewees will be determined based on the data saturation. Participants will be asked at the point of obtaining consent and again, when invited, whether they are comfortable with being contacted to complete the interviews. Because of the COVID-19 pandemic, interviews are likely to be conducted remotely, via telephone or video calls. They will be audio recorded and will last for a maximum of 45 minutes.

Additional facilitator and service staff interviews will also be conducted remotely and audio recorded. An email to the research sites will ask volunteers to complete short interviews, which will discuss their facilitation experiences. Through snowball sampling, we aim to interview 5 additional service staff members who could provide feedback on the potential implementation and maintenance of the PRIDE app intervention. Both interviews will last for a maximum of 30 minutes.

The research team developed semistructured interview schedules. This approach will be adopted to ensure that topics relevant to the study’s aims are discussed. Consideration will be given as to how the order of questioning could improve the interview content and whether prompts will be needed to further expand the answers provided. However, the interview schedule will be flexible enough to allow for the discussion of any additional topics mentioned by the participants, which may be beneficial to the research aims if explored.

For participant interviews, questions will explore their quality of life, experiences of using the intervention, and the impact of the lifestyle changes encouraged by the intervention. The themes covered will include the following:

1. Acceptability of the intervention and whether they enjoyed using the PRIDE app
2. Experience of using the intervention and its impact on daily life
3. Factors that may mediate or moderate the impact or effectiveness of the intervention
4. Likelihood of using the skills or behavior changes in the future
5. Barriers to and facilitators for continued use of the behavior changes encouraged through the intervention

For facilitator interviews, the themes covered will include the following:

1. Barriers to and facilitators for the delivery of the intervention
2. Skills and competencies required for delivery
3. Ease of delivery

For clinical staff, the themes covered will include the following:

1. How the PRIDE app intervention could fit into the existing care model
2. Would it be a financially viable intervention in the current health care system

### Data Analysis

Quantitative and qualitative data will be analyzed to provide insight into whether participants have adopted the intervention in their daily lives, whether they would be willing to continue to use the intervention, and whether it has had a positive effect on their quality of life and dementia self-management. Data will be analyzed anonymously using Stata 17 (StataCorp). As patients will be recruited from various study sites and measured at baseline and follow-up, all measures will be summarized by site and across the measuring time. Outcome data will first be explored through descriptive analysis, with the mean (SD) for normally distributed variables, median (IQR) for skewed variables, and frequency (%) for each level of categorical variables.

To evaluate the efficacy of the PRIDE app, multilevel linear regression modeling will be conducted to quantify the change estimates (95% CI) from baseline to the first and second follow-ups for normally distributed outcomes. The skewed outcome, if any, will be transformed for multilevel modeling. To understand the reach of the PRIDE app, analyses will be conducted on eligibility percentage—the number of potentially eligible participants approached, participation rate, and demographics—to understand who was approached and how representative the final participant sample is. Participant retention rate figures will show how well the intervention was adopted by participants and whether the PRIDE app could be a suitable long-term intervention for people with dementia. Analyses of pre- and postintervention outcome measures will reveal whether the PRIDE app was effective in improving the respective dimensions measured.

From the PRIDE app use data, we will be able to analyze the number of times participants accessed the app, which topics were most popular, and the duration of app use (using log-in and log-off times). These figures will help us understand whether participants actively engaged with the PRIDE app and how well the app was adopted in their daily lives. Missing outcome information will be examined, and its influence on each change score estimate will be checked using data with missingness imputed using multiple imputations with an analytic model used to impute missingness, assuming the missingness mechanism is Missing-At-Random.

Qualitative interview data will be pseudonymized and transcribed verbatim by an NHS-approved transcription service. Participants’ comments will be anonymized to maintain confidentiality. The data will be analyzed through thematic analysis by a PhD student. Thematic analysis has been chosen because of its flexible application; appropriateness for the study’s methodology and research aims; and ability to identify, examine, and report recurring and unexpected themes found within the interviews [22].

A deductive approach to thematic analysis will be incorporated, thereby enabling more focused analysis, with the themes identified driven by the research aims and topics that need to be explored. The following analytical process will be applied [22]:

- Stage I—familiarization of data: the audio recordings will be transcribed and read multiple times to ensure familiarization. Initial ideas for codes are noted in the margin of the transcript.
- Stage II—generating initial codes: initial ideas will be coded and data extracts relevant to these codes collated.
- Stage III—searching for themes: ideas for themes will be developed in the initial coding and extraction stages. Additional data relevant to these themes will be collected. The study’s research aims will be kept in mind during the development of the themes.
- Stage IV—reviewing themes: a diagram will be created and reviewed, showing the relationship among themes, data extracts, and data as a whole.
- Stage V—defining and naming themes: a further thorough analysis of themes will be conducted, with clear definitions and names developed for each theme.
- Stage VI—producing the report: appropriate codes, themes, and data extracts will be finalized for analysis, with these suited to the research aims.

### Monitoring

The occurrence of an adverse event as a result of participation in this study is not expected and therefore will not be routinely recorded by the UoN team. However, individual sites will be able to follow local procedures to monitor and record any events. The UoN team will be informed of any adverse events affecting the study participants.

## Results

The analysis of measures will explore the impact of the PRIDE app on participants’ independence, mood, and quality of life. Pre- and postscores on outcome measures will show any statistical result of the potential effect of participation on individuals. Overall mean scores will help provide insight into the impact of app use across all participants and supporters, providing an indication of whether the PRIDE app could benefit people living with mild dementia and their supporters. With regard to the RE-AIM elements, reach will be understood through the participation rate and demographics, which will show the characteristics of the participants recruited and how well they have been retained. Pre- and postoutcome scores will support potential effectiveness. Adoption will be explored using the participant retention rate and use data gathered from the PRIDE app. This will help us understand whether the participants actively engaged with the app and how well it was adopted in their daily lives.

Interview data will discuss participants’ experiences of taking part in the study, whether they enjoyed using the PRIDE app, and if they felt it had had a positive effect on their well-being and independence. The questions for the facilitator and service staff will focus on the ease of session delivery, barriers to successful delivery, and whether the PRIDE app could be implemented and maintained within the existing health care system. Themes that are generated through the thematic analysis process [22] will complement the quantitative data in terms of the RE-AIM elements, in particular, the adoption, implementation, and maintenance of the PRIDE app by participants and dementia services. Data collection began in June 2021 and is predicted to cease by the end of August 2022. As of January 2022, the study has recruited 4 NHS sites and 23 participants and supporters. Data analysis is yet to begin, and the study findings are anticipated to be published in Spring 2023. All data will be analyzed anonymously.

## Discussion

### Overview

This RE-AIM study will explore the PRIDE app psychosocial intervention to support self-management in people living with mild dementia. Through quantitative and qualitative data, we will evaluate its reach, effectiveness, and adoptability in the independence and quality of life of the participants and their supporters before and after the intervention. Additional data collected from intervention facilitators and clinical staff will help us to better understand how the PRIDE app could be successfully implemented and maintained in existing dementia services.

In some cases, the process of seeking a diagnosis can be prolonged due to service delivery, diagnosis stigma, and more recently, the impact of the COVID-19 pandemic. Regarding the PRIDE app study, this might mean that by the time of diagnosis, some individuals would be ineligible to participate. Therefore, the inclusion criteria ask for mild dementia but place no exact assessment figures. All potential participants will complete a prescreening interview where the relevant researcher will access their suitability and complete a case report form. We recognize that completing measures remotely may result in feelings of embarrassment or reluctance if participants experience issues and do not feel confident about asking for support. However, steps will be taken to provide as much support as possible to the participants throughout their involvement in the study. This will include follow-up contact if measures have not been completed within the timeframe to ensure that participants are not experiencing any issues. Further research on self-management interventions may benefit from including those with mild cognitive impairment and determining whether they have an effect on individuals’ self-management of the condition and any reduction in the risk of developing dementia.

### Limitations

Our study is small scale, with no control group, which reduces the generalizability and reliability of the findings. A small sample size also means that we are not able to demonstrate the individual needs of different dementias. However, if the results indicate potential feasibility and effectiveness, it will be important to conduct a larger trial with a greater number of participants and a control group to validate any initial findings and explore any differences among dementia diagnoses. A patient and public consultations group will be established to provide ongoing input from people and families living with dementia. Members will provide feedback on interview schedules, dissemination materials, and how best to disseminate the findings to relevant people. A paper discussing the development process of the PRIDE app is in progress and will include the original development and more recent modifications.

### Conclusions

Dementia affects every aspect of an individual’s life. Equipping them with relevant knowledge and support facilitates greater self-management and enables people living with dementia and their families to have a better quality of life. This study will be the first to explore whether the PRIDE app intervention can have a positive impact on the self-management of people living with mild dementia through a pre- and postoutcome study design. The knowledge generated from this RE-AIM study will help with the continuing development of the PRIDE app and other similar interventions and in the design of future studies. The data will also help us understand the potential clinical implications of the PRIDE app and how it might be best integrated into existing services.

## Abbreviations

NHS: National Health Service
PRIDE: Promoting Independence in Dementia
RE-AIM: Reach, Effectiveness, Adoption, Implementation, and Maintenance
SPIRIT: Standard Protocol Items: Recommendations for Interventional Trials
UoN: University of Nottingham
